# Assessment of risk factors for early childhood caries at different ages in Shandong, China and reflections on oral health education: a cross-sectional study

**DOI:** 10.1186/s12903-020-01104-8

**Published:** 2020-05-12

**Authors:** Meng Zhang, Xinyue Zhang, Yuan Zhang, Yanan Li, Chunchun Shao, Shijiang Xiong, Jing Lan, Zhifeng Wang

**Affiliations:** 1 Department of pediatrics dentistry, School and Hospital of Stomatology, Cheeloo College of Medicine, Shandong University & Shandong Key Laboratory of Oral Tissue Regeneration & Shandong Engineering Laboratory for Dental Materials and Oral Tissue Regeneration, No.44-1 Wenhua Road West, Jinan, 250012 Shandong China; 2 Center of Evidence-based Medicine, the Second Hospital of Shandong University, Jinan, China; 3 Department of VIP center, School and Hospital of Stomatology, Cheeloo College of Medicine, Shandong University & Shandong Key Laboratory of Oral Tissue Regeneration & Shandong Engineering Laboratory for Dental Materials and Oral Tissue Regeneration, No.44-1 Wenhua Road West, Jinan, 250012 Shandong China; 4 Department of Prosthodontics, School and Hospital of Stomatology, Cheeloo College of Medicine, Shandong University & Shandong Key Laboratory of Oral Tissue Regeneration & Shandong Engineering Laboratory for Dental Materials and Oral Tissue Regeneration, No.44-1 Wenhua Road West, Jinan, 250012 Shandong China

**Keywords:** Dental caries, Health education, Dental, Risk factor, Health knowledge, Attitudes, Practice

## Abstract

**Background:**

The high prevalence of early childhood caries (ECC) is widespread around the world, and oral health education (OHE) plays a vital role in preventing ECC. Numerous studies on ECC risk factor assessment have assisted us in enriching the content of OHE. The objective of this study was to further assess independent risk factors for ECC at different ages to provide evidence and insights for OHE.

**Methods:**

Children aged 3–5 years old (*N* = 1301) in Shandong Province were enrolled in this cross-sectional study. Data about oral health status and caregivers’ oral health knowledge, attitude, and practice (KAP) were extracted from the 4th National Oral Health Survey of China. The associations between ECC prevalence and various KAP variables were tested with chi-square tests, bivariate analysis and multivariable logistic regression analyses.

**Results:**

The ECC prevalence in Shandong Province was 64.6%, and the dmft mean was 3.15. The independent variables with an increased risk for ECC were age, feeding method within 6 months of birth, bedtime sugar frequency, experience of toothache over the past year and dental visits (*P* < 0.05, chi-square tests). Complete breastfeeding within 6 months of birth primarily contributed to the high ECC risk of the 3-year-old group (OR: 3.39, 95% CI: 1.41–8.17), while high frequency bedtime sweet consumption mainly contributed to that of the 5-year-old group (OR: 3.22, 95% CI: 1.03–10.06; logistic regression analysis). Tooth brushing was not associated with ECC in this study, and some positive knowledge and attitude variables were positively correlated with a high risk of ECC.

**Conclusion:**

These data provide evidence to suggest that the ECC-related risk factors at different ages are inconsistent, which provides some insights for OHE. We should highlight the effects of feeding methods in the early stages of deciduous dentition and sugar habits in the late stages of deciduous dentition on ECC, as well as encourage preventive dental visit and supplemental training for oral health practices.

## Background

The high prevalence of early childhood caries (ECC) is a common phenomenon both in China and worldwide [1], coupled with its related adverse effects on oral health and even general health [2]. Hence, strengthening the prevention of ECC has been the most urgent and significant task for both dentists and society as a whole [3].

The current prevention of caries is mainly achieved through the following two important aspects: i) professional preventive measures implemented in dental clinics, including fluoride coating, pit and fissure sealing, etc. [4, 5]; and research involving novel anti-caries materials is one of the hotspots of current basic research; and ii) self-oral health care (S-OHC) behaviour, which, in addition to brushing, gargling, and flossing to remove dental plaque, refers to the control of high-risk factors related to caries, such as sugar consumption and feeding habits [4, 6]. Access to this information is primarily achieved through oral health education (OHE), which can take advantage of platforms such as dental clinic, school, community and network as a medium [7].

OHE has always contributed to dental caries prevention [8, 9], as it brings intuitive guidance and assistance to human beings. OHE details are mainly based on studies involving ECC risk factor assessment, which could provide strong evidence and support for OHE. At present, many high-risk factors associated with dental caries have been demonstrated, such as poverty, race, eating habits, family economic status, education level, etc. [10–12]. However, few studies have separately analysed and assessed the risk factors for each age group; instead, they have considered all preschool children as a whole, which has hindered us from preventing dental caries more effectively by optimizing and enriching OHE.

Shandong Province is located in the lower reaches of the Yellow River and is bordered by the Bohai Sea in the north and the Yellow Sea in the east. The local residents primarily consume wheat and its products. The province’s GDP ranks third in China, and it is one of the representative provinces in northern China. However, few studies have reported the oral health status of children in Shandong Province [13].

Therefore, this cross-sectional study is the first to report and analyse the oral health status and independent risk factors for children aged 3–5 years old in Shandong Province, China, and to pay special attention to the risk factors associated with ECC in different age groups. Those variables involving sociodemographic factors, caregivers’ knowledge, attitude, practice variables and children’s oral health status were collected for statistical analyses.

## Methods

This cross-sectional study was conducted in 2015–2016 in Shandong Province and was part of the 4th National Oral Health Survey in China; it has been approved by the ethics committee of the Chinese Stomatological Association (NO. 2014–003).

### Sample selection

Three- to five-year-old children living in Shandong Province were our target population. The sample size was determined by the equal allocation of the national plan, according to the following calculating formula:

$$ n= deff\frac{u_{\alpha }/{2}^2\ }{\delta^2}p\left(1-p\right) $$

Based on essential caries prevalence rate (*P* = 66.0%, data from the 3rd National Oral Health Survey, 2005), the design effect (deff = 4.5), the significance level (α = 5%), the margin of error (δ = 10%) and the non-response rate (20%), a total of 13,392 children should be recruited for each age group in 31 provinces across the country [14]. According to this at least 1296 sample data should ultimately be enrolled in this study. We incorporated the sample population according to the following multistage stratified sampling method:

1. The probability proportion to size (PPS) sampling method determined the two districts and two counties to be used for the survey.
2. Three kindergartens were randomly selected from each region.
3. Using cluster sampling and quota sampling, 36 children were selected to represent each age group from each kindergarten.

### Examiners selection and training

Three examiners from the Hospital of Stomatology were selected and trained. To ensure the consistency of the clinical practice inter- and intra-examiners, each examiner randomly enrolled three participants for examination. Before calculating the Kappa value, every examination result had to be calibrated with a qualified examiner. The mean Kappa value of the intra-examiners was determined through a re-examination of the samples, and the mean Kappa value of the inter-examiners was calculated through the cross-examination of the samples. Clinical practice training ended when the Kappa value was greater than 0.8.

### Informed consent and questionnaire

After the caregivers had signed the informed consent forms, the children were scheduled for a standard oral examination. In addition, a questionnaire was completed by the caregivers, and then the staff rechecked it for missing items, abnormal items, etc. The questionnaire contents mainly included the following:

1. Personal and family information.
2. OHC knowledge, mainly involved in the effect of brushing, bacteria, sugar, pit and fissure sealing, fluoride and other factors on the teeth and gums.
3. OHC attitude, mainly to investigate whether the caregiver believes that oral health is extremely important.
4. Caregiver-guided OHC practices for children, which mainly obtained information on the important risk factors that have been confirmed by other studies, including feeding methods, sugar consumption, brushing, toothaches, fluoride exposure, dental visits and other detailed information.

### Caries assessment

A ball-end community periodontal index probe was used to assess dental status, according to the WHO guidelines [15]. Individuals who were recorded as having decayed teeth, filled teeth that still had caries, or missing teeth due to caries were included in the ECC group, and the individuals without decayed teeth were included in the caries-free group (CF). In addition, the written version of the examination results and treatment recommendations were sent to the children’s caregivers to increase the significance of the survey.

### Statistical analysis

IBM SPSS Statistics version 17.0 was used to perform the statistical analyses. The prevalence of preschool children with ECC and the mean dmft count were calculated to assess the association of caries with selected KAP variables. Analyses of variance, chi-square tests, Fisher’s test and *z* tests for post hoc comparisons were performed to assess the statistical significance between different variables, all of which were analyzed using the caries-free group as a control. Multivariate logistic regression models (method: backward) were carried out based on the outcomes of ECC (any versus none) to evaluate the independent risk factors while also controlling for latent confounders found in the bivariate analyses (*P* ≤ 0.10). All KAP variables included in the models were categorical variables. Some count variables were recoded into categorical variables if necessary, such as the income variable. Differences were considered significant at *P <* 0.05.

## Results

A total of 2 districts and 2 counties were selected in Shandong Province. In total, 1330 preschool children were invited to participate in the study, but due to the loss of 29 individuals’ information, a total of 1301 children’s data were included in the final statistical analyses. Up to 64.6% of children aged 3–5 had ECC, and the mean dmft (standard deviation) was 3.15 ± 3.845. The ECC rates of the 3-, 4-, and 5-year-old age groups were 51.1, 67.8, and 73.9%, respectively, and the mean dmft values were 2.16 ± 3.096, 3.21 ± 3.966, and 4.01 ± 4.134, respectively (see Table 1), while 96.4% decayed teeth (*n* = 810) in ECC-group remained to be treated.

**Table 1:** Distribution and statistical significance by demographic variables for ECC among a sample of 3–5 years old children in China (*n* = 1301)

Variables	CF (n, (%))	ECC (n, (%))	*P*
gender			
male	235 (36.1)	416 (63.9)	0.616
female	226 (34.8)	424 (65.2)	
Age			
3	203 (48.9)^a^	212 (51.1)^a^	0.000
4	142 (32.2)^b^	299 (67.8)^b^	
5	116 (26.1)^b^	329 (73.9)^c^	
Region			
urban	227 (34.9)	424 (65.1)	0.343
weihai	111 (34.3)	213 (65.7)	
hedong, linyi	116 (35.5)	211 (64.5)	
rural	234 (36.0)	416 (64.0)	
weifang	106 (32.7)	218 (67.3)	
pingyi, linyi	128 (39.3)	198 (60.7)	
Education			
Elementary school and below	88 (34.9)	164 (19.5)	0.699
junior high school	192 (36.8)	330 (39.3)	
Senior high school and above	181 (34.3)	346 (41.2)	
Income			
less than 30,000	111 (34.0)	215 (27.4)	0.357
40,000–60,000	166 (38.0)	271 (34.5)	
more than 70,000	153 (33.8)	300 (38.2)	

*CF* caries-free group, *ECC* Early Childhood Caries group

^a^,^b^, ^c^: Differences of caries prevalence between row variables, the same mark represents no difference between the two variables

Statistical methods: chi-square tests, Fisher’s test and *z* tests for post hoc comparisons

### Statistical analyses of ECC-related variables

The demographic characteristics are shown in Table 1. The ECC rates increased with age (*P* < 0.001), whereas gender, region, education background and income had no association with the rates of ECC in this study.

The distribution characteristics of the KAP variables are shown in Appendix Table S1. A total of 1052 questionnaires (80.9%) were completed by the parents, and the rest were completed by other people (*P* > 0.05, data not shown). From the perspective of OHC knowledge (Q22a-h) and attitude (Q21a-f), 66.0–98.8% of the individuals answered most of the questions correctly. While only 30.7% reported knowing that gum bleeding is not a normal phenomenon when brushing teeth (Q22a), approximately 16.0 and 27.7% reported knowing that pit and fissure sealing or fluoride, respectively, can protect teeth (Q22g, h), and 35.8% believed that the mother’s oral health affects the child’s oral health (21f). The children of those who reported knowing that bacteria (Q22d) and sugar (Q22e) can induce caries and that pit and fissure sealing can protect teeth (Q22g) and those who believe that teeth health is related to their own protection (Q21c), were more likely to experience ECC (*P <* 0.05). From the perspective of OHC practices (Q3–13), children with completely artificial feeding within 6 months of birth had the lowest rates of ECC compared with other feeding methods (Q3, 45.0% vs 65.3–66.5%, respectively; *P* = 0.005). The higher frequency of eating sugary foods before going to bed at night was associated with increased rates of ECC (Q5, 58.6% vs 66.8% vs 73.8%; *P* = 0.002). In addition, 53.1% of all children (*n* = 691) had tooth-brushing habits, with 98.8% using toothpaste; however, only 8.8% (*n* = 60) reported knowing they were using fluoride toothpaste, and these children had the highest rates of ECC compared with other children who did not know the ingredients of their toothpaste (Q11, *n* = 683, 80.0% vs 62.2%; *P* = 0.016); in addition, the earlier the children started brushing their teeth, the lower was the rate of dental caries (Q7, *n* = 691, *P* = 0.033). Children who had frequent toothaches or discomfort in the past 12 months (Q12) or children who had ever visited a dentist (Q13) generally had a higher caries rate than did children who had never experienced either event (*P* < 0.001). The number of brushing times per day were not associated with an increased number of caries in the current study.

### Statistical analyses of ECC-related variables at different ages

Considering that age was a significant difference variable, the differences in ECC rates across the variables in each age group were analysed to eliminate the potential effects of age. The results of the bivariate analyses for the different ages are shown in Table 2. In the 3-year-old group, children raised by those parents who believed that sugar can induce dental decay, and children who were primarily breastfed within 6 months of birth, who had experienced toothache recently, or who had visited a dentist were more likely to suffer from dental caries (*P* < 0.05). In the 4-year-old group, girls generally had higher rates of caries (72.9% vs 62.5%; *P* = 0.025), and children who had ever had a toothache or a dental visit still had the highest rates of ECC (*P* < 0.05). In the 5-year-old group, the frequency of eating sweets before going to bed, toothache experience, dental visit experience and the more favourable knowledge and attitudes among caregivers regarding bacteria being able to cause dental caries were associated with increased rates of ECC (*P* < 0.05). Overall, having experienced toothache and having had dental visits (Q12 and 13) were both correlated with a high risk of ECC for each age group.

**Table 2:** Differential KAP variables in different age groups (*n* = 1301)

Groups and variables	CF (n, (%))	ECC (n, (%))	*P*
**For 3-year-old group (*n*** **= 415)**			
**Knowledge**			
Q22e Does eat sugar cause dental caries?			
correct answer	160 (46.2)^a^	186 (53.8)^a^	0.017
wrong answer or do not know	43 (62.3)^b^	26 (37.7)^b^	
**Practice**			
Q3_group Feeding method within 6 months of birth			
complete breastfeeding	122 (46.6)^a^	140 (53.4)^a^	0.028
mainly breastfeeding	34 (47.9)^a,b^	37 (52.1)^a,b^	
complete artificial feeding	23 (74.2)^b^	8 (25.8)^b^	
mainly artificial feeding	9 (64.3)^a,b^	5 (35.7)^a,b^	
half breastfeeding and half artificial feeding	15 (40.5)^a,b^	22 (59.5)^a,b^	
Q12 Has your child had a toothache or discomfort in the past 12 months?			
Never	179 (54.4)^a^	150 (45.6)^a^	0.000
Sometimes	11 (18.0)^b^	50 (82.0)^b^	
Often	0 (0)^a,b^	4 (100.0)^a,b^	
Not clear	13 (61.9)^a^	8 (38.1)^a^	
Q13 Did your child go to the hospital to see the teeth?			
Yes	24 (35.8)^a^	43 (64.2)^a^	0.023
Never	179 (51.4)^b^	169 (48.6)^b^	
**For 4-year-old group (*n*** **= 441)**			
Gender			
male	81 (37.5)^a^	135 (62.5)^a^	0.025
female	61 (27.1)^b^	164 (72.9)^b^	
**Practice**			
Q12 Has your child had a toothache or discomfort in the past 12 months?			
Never	130 (38.2)^a^	210 (61.8)^a^	0.000
Sometimes	10 (13.5)^b^	64 (86.5)^b^	
Often	0 (0)^b^	13 (100.0)^b^	
Not clear	2 (14.3)^a, b^	12 (85.7)^a,b^	
Q13 Did your child go to the hospital to see the teeth?			
Yes	14 (18.4)^a^	62 (81.6)^a^	0.004
Never	128 (35.1)^b^	237 (64.9)^b^	
**For 5-year-old group (*n*** **= 445)**			
**Knowledge**			
Q22d Can bacteria cause dental caries?			
correct answer	79 (25.0)^a^	237 (75.0)^a^	0.010
wrong answer	9 (60.0)^b^	6 (40.0)^b^	
do not know	28 (24.6)^a^	86 (75.4)^a^	
**Practice**			
Q5 Frequency of eating sweet before going to bed at night			
often	4 (12.1)^a^	29 (87.9)^a^	0.005
occasionally	56 (22.5)^a^	193 (77.5)^a^	
never	56 (34.4)^b^	107 (65.6)^b^	
Q12 Has your child had a toothache or discomfort in the past 12 months?			
Never	102 (35.3)^a^	187 (64.7)^a^	0.000
Sometimes	10 (7.9)^b^	116 (92.1)^b^	
Often	0 (0)^b^	15 (100)^b^	
Not clear	4 (26.7)^a, b^	11 (73.3)^a,b^	
Q13 Did your child go to the hospital to see the teeth?			
Yes	9 (8.7)^a^	95 (91.3)^a^	0.000
Never	107 (31.4)^b^	234 (68.6)^b^	

*CF* caries-free group, *ECC* Early Childhood Caries group

^a, b^: Differences of caries prevalence between row variables, the same mark represents no difference between the two variables

Statistical methods: chi-square tests, Fisher’s test and *z* tests for post hoc comparisons

### Logistic regression analyses

The results of the multivariable logistic regression analyses for ECC are shown in Table 3. Overall, the association between increasing age and the accumulation of ECC was still significant (OR: 2.44, 95% confidence interval (CI): 1.79–3.31 for the 5-year-old group; OR: 2.07, 95% CI: 1.55–2.78 for the 4-year-old group). Factors independently associated with an augmented risk for ECC included being breastfed completely within 6 months of birth (OR: 2.40, 95% CI: 1.46–3.95), a high frequency of consuming sweets before bedtime (OR: 1.81, 95% CI: 1.09–3.02), having experienced a toothache over the past year (OR: 4.7, 95% CI: 3.11–7.11) and having had dental visits (OR: 1.75, 95% CI: 1.19–2.55). Those families who reported knowing that sugar can induce caries were more likely to experience ECC (OR: 1.49, 95% CI: 1.07–2.06). However, toothbrushing was not associated with increased rates of ECC.

**Table 3:** Multivariable logistic regression model for ECC: children aged 3–5 years old (n = 1301; method: backward)

Variables	OR	95% CI	*P*
age			
5 years old	2.44	1.79–3.31	0.000
4 years old	2.07	1.55–2.78	0.001
3 years old	1.00	NA	NA
22e_group Does eat sugar cause dental caries?			
Yes	1.49	1.07–2.06	0.017
No or do not know	1.00	NA	NA
Q3 Feeding method within 6 months of birth			
mainly breastfeeding	2.05	1.19–3.54	0.010
complete breastfeeding	2.40	1.46–3.95	0.001
mainly artificial feeding	1.95	0.93–4.07	0.077
half breastfeeding and half artificial feeding	2.00	1.11–3.63	0.022
complete artificial feeding	1.00	NA	NA
Q5 Frequency of eating sweet before going to bed at night			
often	1.81	1.09–3.02	0.023
occasionally	1.34	1.03–1.74	0.027
never	1.00	NA	NA
Q12 Does your child have toothache or discomfort within the past 12 months?			
Sometimes or often	4.70	3.11–7.11	0.000
Not clear	1.24	0.67–2.23	0.487
Never	1.00	NA	NA
Q13 Has your child ever seen a dentist?			
Yes	1.75	1.19–2.55	0.004
Never	1.00	NA	NA

*OR* odd rates, *CI* confidence interval, *NA* not applicable

Table 4 shows the results of the multivariable logistic regression analyses used to identify risk factors independently associated with ECC in different age groups. For the 3-year-old group, those with a history of having been completely breastfed within 6 months of birth were more likely to experience ECC compared to those who had been completely artificial fed (OR: 3.39, 95% CI: 1.41–8.17); and children with a history of having experienced toothache over the last year (OR: 5.40, 95% CI: 2.70–10.81) and children raised by those caregivers who believed sugar can cause dental caries (OR: 1.77, 95% CI: 1.02–3.07) were more likely to suffer from dental caries. The independent risk factors for the 4-year-old group only included gender and having had a toothache experience over the past year. For the 5-year-old group, the factors independently associated with an increased risk for ECC included having an increased frequency of bedtime sweet consumption (OR: 3.22, 95% CI: 1.03–10.06), having had a toothache experience over the past year (OR: 4.93, 95% CI: 2.39–10.14) and having had dental visits (OR: 2.54, 95% CI: 1.17–5.53). Brushing was still not an independent risk factor for ECC.

**Table 4:** Multivariable logistic regression model for ECC at different age groups (n = 1301; method: backward)

model 1 (For 3-year-old group; n = 415)			
Variables	OR	95% CI	*P*
22e_group Does eat sugar cause dental caries?			
Yes	1.77	1.02–3.07	0.043
No or do not know	1.00	NA	NA
Q3 Feeding method within 6 months of birth			
mainly breastfeeding	3.09	1.17–8.16	0.023
complete breastfeeding	3.39	1.41–8.17	0.006
mainly artificial feeding	1.97	0.49–7.87	0.336
half breastfeeding and half artificial feeding	4.28	1.44–12.69	0.009
complete artificial feeding	1.00	NA	NA
Q12 Does your child have toothache or discomfort within the past 12 months?			
Sometimes or often	5.40	2.70–10.81	0.000
Not clear	0.70	0.28–1.77	0.454
Never	1.00	NA	NA
**model 2 (**For 4-year-old group; n = 441)			
Variables	OR	95% CI	*P*
gender			
male	0.62	0.41–0.94	0.024
female	1.00	NA	NA
Q12 Does your child have toothache or discomfort within the past 12 months?			
Sometimes or often	4.73	2.36–9.49	0.000
Not clear	3.90	0.85–17.80	0.079
Never	1.00	NA	NA
**model 3 (**For 5-year-old group; n = 445)			
Variables	OR	95% CI	*P*
Q5 Frequency of eating sweet before going to bed at night			
often	3.22	1.03–10.06	0.045
occasionally	1.64	1.03–2.61	0.036
never	NA	1.00	NA
Q12 Does your child have toothache or discomfort within the past 12 months?			
Sometimes or often	4.93	2.39–10.14	0.000
Not clear	1.25	0.38–4.14	0.716
Never	NA	1.00	NA
Q13 Did your child go to the hospital to see the teeth?			
Yes	2.54	1.17–5.53	0.018
Never	NA	1.00	NA

*OR* odd rates, *CI* confidence interval, *NA* not applicable

## Discussion

As a populous province, Shandong Province has a population of nearly 100 million people. The current study collected eligible samples to represent the oral health status of this province and its cover areas with different dietary habits. Ultimately, out of a total of 1330 children aged 3–5 years old, 1301 families provided complete and reliable data information, and the response rate was as high as 97.8%, which is very satisfying.

The caries prevalence rates for 3- to 5-year-old children in Shandong Province were 51.1, 67.8 and 73.9%, respectively, which are all higher than the national levels (50.8, 63.6, and 71.9%, respectively), while the dmft mean and the untreated rate of dental caries are lower than the national data (3.15 vs 3.35; 96.4% vs 96.9%) [14]. Other developing countries in Asia, such as Thailand (age 5: 78.5%) [16], Indonesia (age 0–5: 70.0%) [17], Myanmar (age 5–6: 81.3%) and Cambodia (age 3: 84.9%) [18] all have higher rates of ECC compared to those of Shandong Province. In developed countries such as Japan and Singapore, the ECC rates are down to 44.4 and 49%, respectively [19, 20], which are significantly lower than the worldwide result of 63% obtained by statistics [21]; these countries have also already achieved the WHO’s goal of having less than a 50% prevalence rate of ECC for the year 2020 [22]. These data alert us that there is still a long and hard way to go to reduce the prevalence of ECC in Shandong Province, China.

Age has been evidenced to be an independent risk factor for ECC by many studies [10, 18], and this finding was corroborated in the current study. Most studies, however, evaluate 3- to 5-year-old children as whole, with no study assessing the risk factors for each age. Therefore, we speculated that although the age span used in the current study is small, different ages may have different risk factors. Both the chi-square tests and the logistic regression model in the present study confirm this speculation (Tables 2, 3 and 4). Although different age groups share caries-related risk factors—the experience of toothache over the past year—the increased risk for ECC in children aged 3 is independently associated with the feeding method within 6 months after birth, while that of the 5-year-old children is primarily associated with the frequency of bedtime sweet consumption. This heterogeneity indicates that we should have different priorities in regard to OHE for preschool children of different ages.

The association between feeding method and ECC is intricated and involves a variety of factors, including the frequency, duration, amount, time and formula type of the feeding. Previous studies have reported that breastfeeding and its duration are not associated with an increased risk of ECC [10], yet studies have also found that both prolonged breastfeeding and high-frequency feeding in late infancy augment the risk of having dental caries [23, 24]. Interestingly, Van Palenstein et al. [25] reported that breastfeeding during daylight hours beyond the age of 12 months was not associated with ECC for 25- to 30-month-old infants; rather, the relevant factor is that of breastfeeding at night > 2 times and exposing the child to > 15 min per nocturnal feeding. Although the current study also provides evidence that breastfeeding has a greater adverse impact on ECC than that of artificial feeding, which is similar to the results of national studies completed 10 years ago [26], no more detailed information about the duration, frequency, time, and formula type were collected; thus, it is difficult to analyse and attain those specific factors that may have caused such outcomes. After all, the high cariogenicity of bottle feeding has also been confirmed by many studies [27]. One recent study has contributed evidence for the nonlinear association between breastfeeding duration and ECC and the auxiliary role of fluoridated water in reducing the negative effects of feeding on ECC [28]. This finding reminds us that more relevant variables need to be considered in future studies to elucidate the correlation between feeding methods and ECC.

Frequent exposure to dietary sugar and refined carbohydrates is well recognized to be associated with ECC [29–31], and bedtime sweet consumption is particularly serious [29, 32]. Excessive sugar consumption provides abundant nutrients for supragingival microorganisms and induces acid production, enamel-dentine demineralization, and caries formation [8, 33]. The limitation of sugar consumption has therefore been listed in a declaration that has gained worldwide support [34]. Interestingly, the data from the present study found that the increase in the frequency of bedtime sweet consumption was not an independent risk factor for ECC in children aged 3 or 4, it was only so for children aged 5 years. It is not possible from these data to conclude that there is no need to pay attention to the sugar consumption habits of those under 4 years old because sweet preference has been evidenced to be a high risk factor for ECC in children under 23 months of age in Japan [32]. Leroy et al. [35] and Schluter et al. [36] also confirmed that bedtime sweet consumption is significantly associated with ECC for children aged 3 and 4. Accordingly, it is possible that the outcomes of previous undesirable sugar consumption habits manifested in those 5 years old in the current study and thus seen to be an independent risk factor for the current study sample. This is in full compliance with the actions of time factors in the pathogenesis of dental caries [8]. Therefore, even though sugar consumption habits in this study were only risk factors for the 5-year-old age group, knowledge transmission and behavioural interventions should be carried out early in life.

Previous studies have found that infrequent dental visits were correlated with an augmented risk for untreated caries [37]. Some studies, however, have reported a positive association between the number of dental visits and ECC prevalence [10]. This finding is consistent with the results of the current study. Unfortunately, this result goes against the goal of oral prevention. The design of the dental visit is expected to play a positive and energetic role in caries prevention [38]; however, 51.8% of families in the present study sought help from a dentist for treatment compared with only 7.8% who sought help for prevention. An Australian study has reported that dental visits actually contribute 30.3% to the explanation of inequalities in ECC [12]. This result indicates that our awareness of prevention is still insubstantial and that the purpose of dental visits is incorrectly implemented as more for treatment and rarely for prevention. This definition is one of the priorities that we need to improve.

Toothbrushing has always been considered to be the primary and most effective way to clean plaque microbes and prevent dental caries. Many studies have confirmed the association between toothbrushing and ECC [39, 40]. Some studies have also verified that there is no correlation between these two variables [41, 42], which is consistent with the results of the present study. Abdelaziz et al. [43] reported that brushing was not an independent risk factor for ECC and S-ECC in preschool children. Jiang et al. [44] even found that supplemental training in regard to parental toothbrushing would not benefit the reduction of ECC for those children under 3 years of age who lived in a water-fluoridated area and had been provided with sufficient OHE, yet which illustrated the important role that fluoride plays in tooth protection [45]. However, the sure thing is the relationship between the earlier the brushing time and the lower the ECC risk [11, 26], the present study also confirmed this trend through a bivariate analyses. Honestly, the interpretation of these results as being relevant to such issues is further limited due to the deficiency of information about brushing details; for example, brushing duration, brushing compliance, floss assistance, and especially brushing efficiency assessment are not available in our data. It is therefore difficult to objectively assess the association between toothbrushing and ECC in the scenario of many factors being masked or even neglected. However, the special dietary habits of Shandong Province, in which wheat is the main food ingredient, may be one of the reasons why toothbrushing was not demonstrated to be an independent risk factor for ECC. Further studies should include these variables so that we can attain a clearer picture of the role of toothbrushing in ECC prevention.

The results of the present study actually reflect the current status and issues of oral health education in Shandong Province. First, there is a sharp contrast between the high awareness of oral health routine knowledge (66–98.8%) and the low access to oral prevention knowledge (16–35.8%), a negative association between some correct knowledge and attitude variables (Q22d/e: sugar or bacteria can induce ECC; Q21c: dental health is related to self-protection.) and ECC risk (Appendix Table S1, Tables 2, 3 and 4), and a positive correlation between frequency of toothache or dental visits and ECC. All of these results reflect that the knowledge and information provided by the current OHE in Shandong Province appears to be inefficient and that it underperforms in promoting the implementation of ECC prevention practices. However, many studies have confirmed that when a more systematic, proactive oral health intervention involving brushing training, referrals and dental care is implemented for a family with children, even if implemented by nondental professionals [46], it generally has a greater preventive effect against ECC [38, 47, 48]. Therefore, a systematic and sound oral health care programme that involves clinical care, brushing training, referrals and so on is imperative [49], rather than just oral health education. Only in this way can we ensure the consistency between theoretical knowledge and actual action as much as possible, thereby reducing the incidence rates of ECC. In addition, the risk factors for ECC at different ages (3–5 years old) are not completely consistent. In the current study, dental caries in the early stage of deciduous dentition (3 years old) is mainly related to feeding methods, while dental caries in the later stage of deciduous dentition (5 years old) is mainly associated with sugar-eating habits. These outcomes indicate that a more strategic and more targeted OHE method and content should be designed for children of different ages, which would also be more cost-effective. Finally, although a positive association between toothbrushing, the use of fluoride and the low risk of dental decay was not obtained in this study, the role of such an association in caries prevention is indeed indelible [11, 50, 51]. Therefore, assisting children to master this knowledge and these skills early in life is the responsibility of both dentists and parents.

In summary, we hope that these data will provide guidance and evidence for improving and refining the contents of oral health education. Based on the results of this study, we should have different focuses for different age groups. In the early stages of deciduous dentition (less than 3 years old), in addition to routinely introducing oral health knowledge and cultivating oral health awareness, we should emphasize the impact of feeding habits in infancy and early childhood on dental health. It seems inconclusive which feeding method (breastfeeding vs artificial milk feeding) is more beneficial to dental health, but issues such as feeding methods and frequency of night milk consumption deserve to be emphasized. As children get older, the training and mastery of certain oral health behaviours should become a priority, including sugar consumption habits and toothbrushing habits.

The main advantage of this study is its large representative sample of children and its high response rate; also, this study is part of the 4th National Oral Health Survey of China and thus has highly reliable data sources. Furthermore, this study is the first to report the oral health status of children in Shandong Province over the past decade. Limitations of the study include the issues related to the OHC knowledge and attitude items designed for the questionnaire being relatively basic and traditional. Several major risk factors have been incorporated into the current study, unlike the monographic studies that have excavated and explored sufficient detailed information about one certain targeting factor associated with ECC [24, 28, 44]. We also have to acknowledge that some data on detailed information is limited, such as the frequency of bedtime feedings, brushing efficiency, floss assistance status, and cariogenic microbial flora, each of which is reported to be associated with ECC. Therefore, more information is waiting to be collected to further analyse the risk factors associated with ECC for each age group.

## Conclusion

The data in the present study provide evidence that different risk factors are independently associated with an augmented risk for ECC at different ages. Feeding methods primarily contribute to the risk in the early stage of deciduous dentition, while sugar habits mainly contribute to the risk in the late stage of deciduous dentition, which bring some insights for OHE. In addition, preventive dental visit and additional training for oral health practices are encouraged to improve the OHC programme and reduce the ECC rates.

## Supplementary information

**Additional file 1: Table S1.**. Distribution and statistical significance by guardians’ knowledge, attitude, and behavioral variables for ECC among a sample of 3–5 years old children in China (*n* = 1301).

## Data Availability

The datasets used and analyzed during the current study are available from the corresponding author on reasonable request.

## Abbreviations

ECC: Early childhood caries
CF: Caries-free
KAP: Knowledge, attitude and practice
OHC: Oral health care
OHE: Oral health education
WHO: World health organization
dmft: decayed, missing and filled teeth
RCT: Randomized controlled trial
OR: Odd rates
CI: Confidence interval
