# Supplementary material for: Assessment of risk factors for early childhood caries at different ages in Shandong, China and reflections on oral health education: a cross-sectional study
Source: BMC Oral Health. 2020 May 12;20:139. doi: 10.1186/s12903-020-01104-8 (PMC7218629; doi:10.1186/s12903-020-01104-8)
Supplement: Supplementary file 1 — Additional file 1: Table S1.. Distribution and statistical significance by guardians’ knowledge, attitude, and behavioral variables for ECC among a sample of 3–5 years old children in China (n = 1301). [file 12903_2020_1104_MOESM1_ESM.docx]

Table S1. Distribution and statistical significance by guardians' knowledge, attitude, and behavioral variables for ECC among a sample of 3-5 years old children in China (n=1301).

| Variables | CF (n, (%)) | ECC (n, (%)) | *P* | |
| --- | --- | --- | --- | --- |
| **Knowledge** |  |  |  | |
| Q22a Is gum bleeding normal when brushing? | | | |  |
| correct answer | 76 (33.8) | 149 (66.2) | 0.151 | |
| wrong answer | 312 (34.6) | 590 (65.4) |  |  |
| do not know | 73 (42.0) | 101 (58.0) |  |  |
| Q22b Can bacteria cause inflammation of the gums? | | | |  |
| correct answer | 372 (34.5) | 707 (65.5) | 0.281 | |
| wrong answer | 23 (40.4) | 34 (59.6) |  |  |
| do not know | 66 (40.0) | 99 (60.0) |  |  |
| Q22c Can brushing teeth cause bleeding gums? | | | |  |
| correct answer | 303 (35.3) | 555 (64.7) | 0.673 | |
| wrong answer | 50 (32.9) | 102 (67.1) |  |  |
| do not know | 108 (37.1) | 183 (62.9) |  |  |
| Q22d Can bacteria cause dental caries? | | | |  |
| correct answer | 328 (34.6)^a^ | 619 (65.4)^a^ | 0.026 | |
| wrong answer | 28 (52.8)^a^ | 25 (47.2)^b^ |  |  |
| do not know | 105 (34.9)^a^ | 196 (65.1)^a^ |  |  |
| Q22e Does eating sugar cause dental caries? | | | |  |
| correct answer | 371 (33.8)^a^ | 727 (66.2)^a^ | 0.013 | |
| wrong answer | 32 (41.6)^a,b^ | 45 (58.4) |  |  |
| do not know | 58 (46.0)^b^ | 68 (54.0)^b^ |  |  |
| Q22f Does decayed deciduous teeth need treatment? | | | |  |
| correct answer | 333 (36.1) | 590 (63.9) | 0.606 | |
| wrong answer | 50 (36.0) | 89 (64.0) |  |  |
| do not know | 78 (32.6) | 161 (67.4) |  |  |
| Q22g Can the pit and fissure sealing prevent dental caries? | | | |  |
| correct answer | 60 (28.8)^a^ | 148 (71.2)^a^ | 0.030 | |
| wrong answer | 29 (44.6)^a^ | 36 (55.4)^b^ |  |  |
| do not know | 372 (36.2)^a^ | 656 (63.8)^b^ |  |  |
| Q22h Can fluoride protect teeth? | | | |  |
| correct answer | 118 (32.8) | 242 (67.2) | 0.251 | |
| wrong answer | 29 (42.6) | 39 (57.4) |  |  |
| do not know | 314 (36.0) | 559 (64.0) |  |  |
| **Attitude** | | | |  |
| Q21a_group Is oral health important to your life? | | | |  |
| correct answer | 453 (35.3) | 832 (64.7) | 0.220 | |
| wrong answer | 8 (50) | 8 (50.0) |  |  |
| Q21b_group Is regular oral examination necessary? | | | |  |
| correct answer | 421 (34.8) | 790 (65.2) | 0.064 | |
| wrong answer | 40 (44.4) | 50 (55.6) |  |  |
| Q21c_group Is the quality of your teeth related to your own protection? | | | |  |
| correct answer | 357 (33.6)^a^ | 704 (66.4)^a^ | 0.005 | |
| wrong answer | 104 (43.3)^b^ | 136 (56.7)^b^ |  |  |
| Q21d_group to prevent caries, you must first rely on yourself. | | | |  |
| correct answer | 431 (34.9) | 804 (65.1) | 0.081 | |
| wrong answer | 30 (45.5) | 36 (54.5) |  |  |
| Q21e_group Is it important to protect child's first molar? | | | |  |
| correct answer | 396 (35.1) | 731 (64.9) | 0.569 | |
| wrong answer | 65 (37.4) | 109 (62.6) |  |  |
| Q21f_group Does the mother's unhealthy teeth affect the child's teeth? | | | |  |
| correct answer | 169 (36.3) | 297 (63.7) | 0.639 | |
| wrong answer | 292 (35.0) | 543 (65.0) |  |  |
| **Practice** | | | |  |
| Q3 Feeding method within 6 months of birth | | | |  |
| complete breastfeeding | 256 (33.9)^a^ | 499 (66.1)^a^ | 0.005 | |
| mainly breastfeeding | 88 (33.5)^a^ | 175 (66.5)^a^ |  |  |
| complete artificial feeding | 44 (55.0)^b^ | 36 (45.0)^b^ |  |  |
| mainly artificial feeding | 22 (39.3)^a, b^ | 34 (60.7) |  |  |
| half breastfeeding and half artificial feeding | 51 (34.7)^a^ | 96 (65.3)^a^ |  |  |
| Q4a Frequency of eating sweet food | | | |  |
| rare/never | 105 (41.5) | 148 (58.5) | 0.248 | |
| 1-3 times/month | 60 (33.7) | 118 (66.3) |  |  |
| 1 time/week | 60 (36.4) | 105 (63.6) |  |  |
| 2-6 times/week | 111 (33.3) | 222 (66.7) |  |  |
| 1 time/day | 79 (35.7) | 142 (64.3) |  |  |
| ≥2 times/day | 46 (30.5) | 105 (69.5) |  |  |
| Q4b Frequency of eating sweet drinks | | | |  |
| rare/never | 229 (36.7) | 395 (63.3) | 0.315 | |
| 1-3 times/month | 86 (31.3) | 189 (68.7) |  |  |
| 1 time/week | 54 (40.3) | 80 (59.7) |  |  |
| 2-6 times/week | 48 (32.0) | 102 (68.0) |  |  |
| 1 time/day | 28 (41.2) | 40 (58.8) |  |  |
| ≥2 times/day | 16 (32.0) | 34 (68.0) |  |  |
| Q5 Frequency of eating sweet before going to bed at night | | | |  |
| often | 27 (26.2)^a^ | 76 (73.8)^a^ | 0.002 | |
| occasionally | 252 (33.2)^a^ | 506 (66.8)^a^ |  |  |
| never | 182 (41.4)^b^ | 258 (58.6)^b^ |  |  |
| Q6 Frequency of children brushing | | | |  |
| every day | 244 (35.3) | 447 (64.7) | 0.921 | |
| occasionally or never | 217 (35.6) | 393 (64.4) |  |  |
| Q7 Age at which to start brushing | | | |  |
| < 1 year old | 10 (38.5)^a,b^ | 16 (61.5)^a,b^ | 0.033 | |
| 2 years old | 73 (42.0)^b^ | 101 (58.0)^b^ |  |  |
| 3 years old | 127 (35.0)^a,b^ | 236 (65.0)^a,b^ |  |  |
| 4-5 years old | 31 (25.2)^a^ | 92(74.8)^a^ |  |  |
| do not remember | 3 (60.0)^a,b^ | 2 (40.0)^a,b^ |  |  |
| Q8 Brushing times per day | | | |  |
| 2 times and above | 39 (28.9) | 96 (71.1) | 0.056 | |
| 1 time | 148 (35.0) | 275 (65.0) |  |  |
| not daily brush | 57 (42.9) | 76 (57.1) |  |  |
| Q9 Frequency of helping child brushing | | | |  |
| daily | 27 (31.0) | 60 (69.0) | 0.470 | |
| weekly | 7 (58.3) | 5 (41.7) |  |  |
| sometimes | 58 (34.7) | 109 (56.3) |  |  |
| occasionally | 75 (35.5) | 136 (64.5) |  |  |
| never done | 77 (36.0) | 137 (64.0) |  |  |
| Q10 Whether to use toothpaste | | | |  |
| Yes | 238 (34.8)^a^ | 445 (65.2)^a^ | 0.018 | |
| No or do not know | 6 (75.0)^b^ | 2 (25.0)^b^ |  |  |
| Q11 Whether to use fluoride toothpaste | | | |  |
| Yes | 12 (20.0)^a^ | 48 (80.0)^a^ | 0.016 | |
| No | 52 (31.9)^a, b^ | 111 (68.1) |  |  |
| do not know | 174 (37.8)^b^ | 286 (62.2)^b^ |  |  |
| Q12 Does your child have toothache or discomfort within the past 12 months? | | | | |
| Never | 411 (42.9)^a^ | 547 (57.1)^a^ | 0.000 | |
| Sometimes | 31 (11.9)^b^ | 230 (88.1)^b^ |  |  |
| Often | 0 (0)^b^ | 32 (100.0)^b^ |  |  |
| Not clear | 19 (38.0)^a^ | 31 (62.0)^a^ |  |  |
| Q13 Has your child ever visited a dentist? | | | | |
| Yes | 47 (19)^a^ | 200 (81.0)^a^ | 0.000 | |
| Never | 414 (39.3)^b^ | 640 (60.7)^b^ |  |  |

CF: caries-free group; ECC: Early Childhood Caries group.

^a^,^b^: The difference of caries prevalence between row variables, the same mark represents no difference between the two variables.

Statistical methods: [chi-square test](https://www.sciencedirect.com/topics/medicine-and-dentistry/chi-square-test)s, Fisher’s test and *z* tests for post hoc comparisons.
